# Understanding Suitable Habitats and Anthropogenic Mortality Risks for King Cobras in Nepal

**DOI:** 10.1002/ece3.72030

**Published:** 2025-09-04

**Authors:** Rishi Baral, Binaya Adhikari, Abhisek Sapkota, Saroj Panthi, Chiranjeevi Khanal, Sandesh Gurung, Keshab Raj Sapkota, Naresh Subedi, Bishwanath Rijal, Raju Acharya, Michito Shimozuru, Toshio Tsubota

**Affiliations:** ^1^ Laboratory of Wildlife Biology and Medicine, Department of Environmental Veterinary Science, Graduate School of Veterinary Medicine Hokkaido University Sapporo Japan; ^2^ Snake Conservation Society, Nepal Pokhara Nepal; ^3^ Friends of Nature (FON Nepal) Kathmandu Nepal; ^4^ Department of Biology University of Kentucky Lexington Kentucky USA; ^5^ Institute of Forestry Tribhuvan University Pokhara Nepal; ^6^ Division Forest Office Baglung Nepal; ^7^ Himalayan Raptors Madhyapur Thimi Bagmati Province Nepal; ^8^ National Trust for Nature Conservation Lalitpur Nepal; ^9^ Green Guard‐Nepal Kathmandu Nepal; ^10^ One Health Research Center Hokkaido University Sapporo Japan

**Keywords:** climate projections, conservation prioritization, spatial analysis, species distribution modeling, venomous snakes

## Abstract

The king cobra ( *Ophiophagus hannah* ), the world's largest venomous snake, is a vulnerable species with an expanding distribution in Nepal. This study modeled its current climatically suitable habitat and predicted future changes (2050 and 2070) under the SSP2‐4.5 climate change scenario. Using 553 occurrence records and a combination of climate and topographic variables, we developed an ensemble habitat suitability model in BIOMOD2, identifying 23,702.62 km^2^ of suitable habitat. Under the SSP2‐4.5 scenario, the king cobra's climatically suitable habitat is projected to decline by 22% by 2050, with a relatively lower decline of 9% by 2070, indicating a potential partial shift or recovery over time. Within Nepal's protected areas, the currently suitable habitat of 3088.34 km^2^ is expected to decrease by 14% by 2050 and 13% by 2070, highlighting vulnerabilities even within formally conserved regions. The analysis highlighted Bagmati Province (7311.19 km^2^) and Gandaki Province (5935.10 km^2^) as key regions, with significant habitats in Chitwan National Park and Annapurna Conservation Area. However, most suitable habitats (over 60%) are located outside protected areas, emphasizing the need for effective conservation strategies. The distribution of king cobra habitats was found significantly influenced by precipitation during the warmest quarter. Analysis of 94 king cobra mortality records (2000–2024) across Nepal identified eastern lowland and mid‐hill regions as critical hotspots, highlighting the urgency for focused conservation initiatives in these high‐risk zones. These insights underline the need for urgent conservation measures to protect this species and its rapidly changing habitat under future climate scenarios.

## Graphical abstract

The king cobra ( *Ophiophagus hannah* ), a vulnerable species, faces significant habitat loss and mortality risks in Nepal. Using ensemble modeling with 553 occurrence records, this study identified 23,702.62 km^2^ of suitable habitat, projecting up to 22% habitat loss by 2050 under SSP2‐4.5 climate scenarios. With over 60% of suitable habitats located outside protected areas and eastern lowland and mid‐hill regions identified as mortality hotspots, the findings underscore the need for targeted conservation strategies.

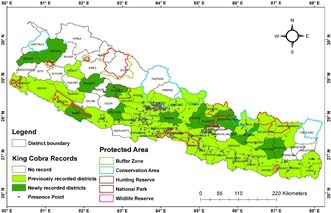

## 1 Introduction

Climate change poses a significant threat to the survival of animal populations worldwide (Masson‐Delmotte et al. 2021). Studies have highlighted various responses of wildlife to increasing temperatures, such as changes in geographic distribution (Pecl et al. 2017) and the timing of life events (Inouye 2022) at the population level, along with behavioral adaptations observed in individuals (Cunningham et al. 2021). Climate change and biodiversity loss stand as two of the most critical challenges facing the planet, driven by rapid urbanization, industrialization, and growing human populations (Lee et al. 2023; Urban 2015). These pressures have led to widespread habitat destruction through deforestation, land‐use changes, and ecosystem fragmentation, severely endangering the survival of countless species (Powers and Jetz 2019; Tucker et al. 2018). Many regions, including Nepal, face mounting extinction risks fueled by climate change, invasive species, habitat degradation, and other human‐induced impacts (Adhikari, Bhandari, et al. 2022; Baral et al. 2024). Recognizing these escalating threats, researchers and policymakers have prioritized understanding biodiversity's vulnerability to guide conservation efforts (Pacifici et al. 2015; Pereira et al. 2010). In this context, tools like Ecological Niche Modeling (ENM) have emerged as powerful methods to predict species distributions by integrating biotic and abiotic factors under various climate scenarios (Sillero et al. 2021; Soberon and Peterson 2005). ENM provides essential insights into habitat connectivity, vulnerability, and the potential impacts of climate change, aiding in effective conservation planning and the mitigation of human–wildlife conflicts (Khosravi et al. 2022).

The king cobra, *Ophiophagus hannah* (Cantor 1836), lies in the monotypic genus of the family Elapidae, which is considered a species complex (Das 2002). This species is the largest venomous snake in the world, which is capable of attaining a length of 4.8–6 m (Campden‐Main 1970; Daniel 1983; Schmidt and Inger 1957; Whitakar and Captain 2008; Zug 1993). This species is widespread globally from Bali, Bangladesh, Bhutan, Myanmar, Nepal, Macao, Indonesia, Hong Kong, Borneo, Cambodia, China, India, Java, Laos, Malaysia, Philippines, Singapore, Sulawesi, Sumatra, Vietnam, and Thailand in South and Southeast Asia (David and Vogel 1996; Iskandar and Colijin 2001; Schleich and Kästle 2002). Recent taxonomic revisions have expanded the diversity of king cobras (genus *Ophiophagus*), introducing new species alongside the classic *Ophiophagus hannah* , which remains distributed across eastern Pakistan, India, central Thailand, Indo‐Burma, Indo‐China, and the Andaman Islands (Das et al. 2024). The name *Ophiophagus bungarus* has been revived for populations in the Sunda Shelf region, including the Malay Peninsula, Greater Sunda Islands, and parts of the southern Philippines. Additionally, two newly described species have been identified: *Ophiophagus kaalinga*, endemic to the Western Ghats of southwestern India, and *Ophiophagus salvatana*, restricted to Luzon Island in the Philippines (Das et al. 2024). This species is listed as vulnerable in the IUCN Red List of Threatened Species and in Appendix II of CITES (CITES 2019; Stuart et al. 2012b). This species is considered rare in Nepal and has been listed as Vulnerable in the National Red Data Book of Nepal since 1995 (Suwal et al. 1995).

The King cobra is a diurnal species which is found to be an inhabitant of dense forests and their surrounding areas in the rural agricultural lands (Shah 2000). This species has several distinct patterns and color morphs (Scott 1995). King cobras have been recorded throughout Terai, within altitudes of 110–2500 m, but are suspected to occur even higher (Shah 2000). It was recorded for the first time in Nepal from Chitwan and Rautahat districts (Fleming Jr. and Fleming Sr. 1974). The old record of this species has been noted from 42 districts (21 districts of Terai and Inner Terai, and 21 district of hilly region) of the country (Devkota et al. 2020, 2021; Rai 2020; Rawat et al. 2020; Baral, Subedi, and Yadav 2019; Baral, Yadav, et al. 2019; Shah 2000; Thapa et al. 2019). King cobras typically nest and reproduce at elevations of 1000–1500 m, but they face growing threats from habitat destruction, human persecution, illegal trade, and the impacts of climate change (Sapkota et al. 2021).

This species lives in dense forest, where deforestation led their original habitats to cultivation land (Shah 2000). King cobras dwell in a home range of 19–710 ha and are frequently observed in the forest edges (Jones et al. 2020; Marshall et al. 2019, 2020). King cobras mostly dwell close to water sources in the edges of the forest and prefer to build nests in areas with an average leaf‐litter height of 10–17 cm (Rao et al. 2013). Mobility is hindered by agricultural practice and other human activities (Marshall et al. 2020). The studies concerning the distribution of king cobras, suitable habitat, human–king cobra conflict, and its conservation implications are very limited. The topographical variation and existing vegetation diversity may have made Nepal one of the favorable habitats for king cobras too. This publication gives the suitable habitat of king cobras in Nepal. This study's goals were to (a) determine relevant influencing bioclimatic variables in the target landscape, (b) predict potential distribution of king cobra habitat across the country, (c) future distribution and range change, and (d) anthropogenic mortality and trends of conflicts for future effective conservation implementation. These findings may provide insight into king cobra habitat protection nationally and help mitigate snake bite incidents.

## 2 Materials and Methods

### 2.1 Study Area

**FIGURE 1:**
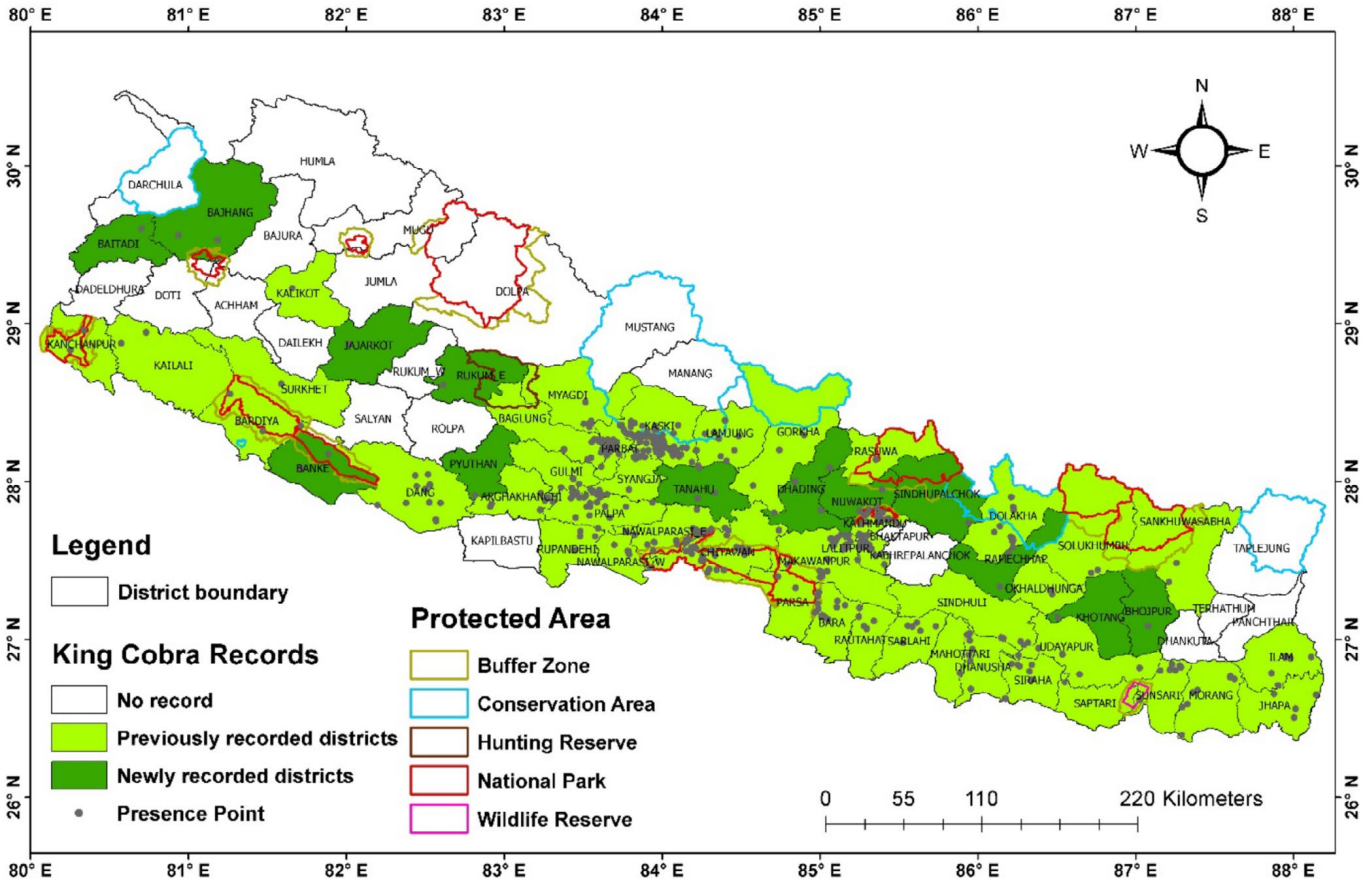
Map of the study area with protected area boundary and king cobra recorded districts with presence points.

Nepal, a central Himalayan nation spanning 147,516 km^2^, is renowned for its extraordinary biodiversity shaped by dramatic topography and climatic variation (Baral et al. 2024; Kunwar et al. 2023; Paudel et al. 2018) Situated between 26.36° N to 30.45° N and 80.06° E to 88.2° E (Figure 1), its elevation ranges from 59 m in the Terai to 8848.86 m at Mount Everest, fostering ecosystems from tropical lowlands to alpine tundra (Bhuju et al. 2007). Rainfall varies significantly, from 271.4 mm in Jomsom to 5514.7 mm in Lumle, while temperatures range between 10.9°C and 24.6°C (Ichiyanagi et al. 2007). The country's geography is divided into five zones. The Terai, below 300 m, features tropical forests with *Shorea robusta* and *Acacia catechu* . The Siwalik (301–1000 m) hosts subtropical species like *Alnus nepalensis* . The Mid‐Hills (1000–3000 m) transition from subtropical to temperate climates with *Rhododendron* spp. and *Alnus*. The High Mountains (3000–5000 m) have cold temperate conditions dominated by *Pinus* and *Rhododendron*, while the High Himalayas (above 5000 m) support alpine scrub and tundra, including *Juniperus*‐*Rhododendron* associations (Baral et al. 2024; Malla et al. 2023; Uddin et al. 2015).

Designated as a global biodiversity hotspot, Nepal supports 3.2% of the world's plant species and 1.1% of its animal species (DNPWC and DFSC 2022; Myers et al. 2000; Poudel et al. 2016; Shrestha and Bajracharya 2018). Of 212 mammal species, 23% are nationally threatened, including 9 critically endangered species (Amin et al. 2018; Baral, Subedi, and Yadav 2019). Nepal harbors 89 species of snakes, of which about 20 are venomous, including cobras, kraits, and pit vipers, such as the king cobra ( *Ophiophagus hannah* ), Monocled cobra ( *Naja kaouthia* ), spectacled cobra ( *Naja naja* ), common krait ( *Bungarus caeruleus* ), banded krait ( *Bungarus fasciatus* ), Himalayan pit viper ( *Gloydius himalayanus* ), Mountain pit viper ( *Ovophis monticola* ), Russell's viper ( *Daboia russelii* ), Green pit viper ( *Trimeresurus albolabris* ), and other *Trimeresurus* spp. (Joshi 2024; Rai et al. 2022; Schleich and Kästle 2002). These snakes play vital roles in regulating prey populations and maintaining ecosystem balance. Protected areas cover 23.39% of the land, comprising 12 national parks, 6 conservation areas, and other reserves, safeguarding species like the king cobra ( *Ophiophagus hannah* ) (Adhikari, Baral, et al. 2022; Baral et al. 2024; DNPWC and DFSC 2022). With its rich ecological and cultural diversity, Nepal's unique landscape remains a critical area for biodiversity conservation and research, extending from tropical plains to the world's highest peaks (Bhuju et al. 2007; DNPWC 2019).

### 2.2 Occurrence Data Collection and Filtering

We compiled two distinct datasets of king cobra ( *Ophiophagus hannah* ) occurrence records, categorized as first‐hand and second‐hand sources. First‐hand data were obtained between 2015 and 2024, primarily through systematic rescue surveys conducted by the Snake Conservation Society Nepal (SCSN). These data were further supplemented by verified sightings and incident reports shared via digital platforms, including Facebook, Twitter, LinkedIn, YouTube, and online news articles. The year 2015 marks the formal initiation of snake rescue and conservation activities by SCSN—the first organization in Nepal dedicated to such work. Second‐hand data, covering the period from 2005 to 2024, were collected from a variety of published and unpublished sources. These included peer‐reviewed research articles, gray literature, and reports issued by government and nongovernmental organizations such as the Department of National Parks and Wildlife Conservation (DNPWC), Department of Forest and Soil Conservation (DoFSC), Division Forest Offices (DFOs), and various Protected Areas (PAs). The year 2005 was selected as the starting point for historical records, as it marks the earliest documented presence of king cobras in Nepal.

In total, we identified 553 occurrence records, encompassing a range of events such as rescues, nest observations, mortalities, and releases. Notable field data were collected from the districts of Kaski, Parbat, Syangja, and Palpa. Supporting literature included works such as Baral, Subedi, and Yadav (2019); Baral, Yadav, et al. (2019); Baral and Koirala (2008); Devkota et al. (2020, 2021); Rai (2020); Rawat et al. (2020); Shah (2000); Thapa et al. (2019). To ensure robustness and minimize redundancy, we implemented a rigorous data cleaning process. Inclusion criteria required complete metadata—specifically, date, location, and supporting evidence (e.g., photographs or videos). Records were cross‐verified through direct communication with rescuers and comparison across sources (e.g., overlapping reports on social media and news outlets). Duplicates were excluded based on matching date, time, and spatial information. Records lacking key metadata, unverifiable identity, or lacking supporting evidence were excluded.

To minimize spatial autocorrelation—the tendency for nearby occurrences to share both the same environment and the same observation bias—we applied the *SpThin* package in R (Aiello‐Lammens et al. 2015; R Core Team 2021). Spatial autocorrelation violates the independence assumption of most SDM algorithms and can inflate model fit statistics and over‐emphasize heavily sampled areas, leading to overoptimistic projections (Kramer‐Schadt et al. 2013). *SpThin* iteratively removes records that fall within a user‐defined distance of one another; we set this distance to 1 × 1 km, retaining a single representative point per grid cell. The procedure reduced the original 553 records to 225 spatially independent localities, thereby preventing pseudoreplication, reducing overfitting, and improving the generalizability of the ensemble models (Boria et al. 2014).

### 2.3 Climatic and Topographic Data

To characterize climatic suitability, we used 19 BIOCLIM indices and three terrain variables—elevation, slope, and aspect (Table 1)—sourced from WorldClim v2.1 (Fick and Hijmans 2017). These layers summarize 1970–2000 climate conditions at a 30 arc‐second (~1 km) resolution and provide biologically meaningful metrics of annual averages, seasonality, climatic extremes, and quarterly heat–moisture balances. Together, they capture key environmental gradients—such as temperature, precipitation, and microclimate—that are known to influence king cobra physiology, prey availability, and den‐site selection. All rasters were resampled to 30 arc‐second resolution (~1 km^2^) and the nine predictors with a variance inflation factor < 3 were retained to minimize multicollinearity (Zuur et al. 2010). Future conditions were projected with the CMIP6 BCCCSM2MR grids under SSP24.5 for 2050 and 2070. We selected this “middleoftheroad” pathway because it closely matches the median trajectory implied by current national emissions pledges, sits between the optimistic SSP12.6 and pessimistic SSP58.5, and is therefore widely used as a policy‐relevant baseline in biodiversity assessments (Hausfather and Peters 2020; Schwalm et al. 2020) as this moderate emissions trajectory provides a pragmatic yet policy‐relevant signal while keeping computational demands tractable. Restricting the analysis to this single, policy‐relevant baseline balanced scientific rigor with computational tractability—avoiding hundreds of additional algorithm × GCM × SSP runs—and allowed us to keep equal emphasis on our second objective, mapping present‐day anthropogenic mortality risk, so that climate projections and risk hotspots could be interpreted together within a practical conservation framework.

**TABLE 1:** Descriptions of bioclimatic and topographic variables used for ecological niche modeling of suitable habitats for the king cobra ( *Ophiophagus hannah* ) in Nepal.

Data sources	Categories	Variables	Abbreviation	Units
WorldClim	Bioclimatic (version 2)	Annual mean temperature	bio1	°C
		Mean diurnal range (mean of monthly [max temp–min temp])	bio2	°C
		Isothermality (BIO2/BIO7)	bio3	Dimensionless
		Temperature seasonality (standard deviation)	bio4	°C
		Max temperature of warmest month	bio5	°C
		Min temperature of coldest month	bio6	°C
		Temperature annual range (BIO5‐BIO6)	bio7	°C
		Mean temperature of wettest quarter	bio8	°C
		Mean temperature of driest quarter	bio9	°C
		Mean temperature of warmest quarter	bio10	°C
		Mean temperature of coldest quarter	bio11	°C
		Annual precipitation	bio12	mm
		Precipitation of wettest month	bio13	mm
		Precipitation of driest month	bio14	mm
		Precipitation seasonality (coefficient of variation)	bio15	Dimensionless
		Precipitation of wettest quarter	bio16	mm
		Precipitation of driest quarter	bio17	mm
		Precipitation of warmest quarter	bio18	mm
		Precipitation of coldest quarter	bio19	mm
USGS GTOPO30	Topographic	Elevation	Elevation	m
		Aspect	Aspect	Degree
		Slope	Slope	Degree

*Note:* This table lists the climate and topographic variables obtained from WorldClim and USGS GTOPO30 datasets, which were used as predictors in species distribution modeling to identify current and future suitable habitats for king cobras.

### 2.4 Biological Significance of Environmental Variables

Bioclimatic variables represent biologically meaningful aspects of temperature and precipitation that are known to influence species' physiology, behavior, and habitat preferences (Elith and Leathwick 2009; Guisan et al. 2007; Precht et al. 1973; Sutton et al. 2017). For example, variables like annual mean temperature (bio1), precipitation of the driest quarter (bio17), and temperature seasonality (bio4) help capture extreme environmental conditions and variability that may limit species distributions (Kearney and Porter 2009; Sunday et al. 2011). These factors can affect thermoregulation, prey availability, and breeding success, which are critical for ectothermic species such as the king cobra (Sapkota et al. 2021; Sutton et al. 2017). Topographic variables—elevation, slope, and aspect—were included to account for microhabitat heterogeneity (Guisan and Zimmermann 2000; Peterson et al. 2011). Elevation influences temperature and moisture gradients; slope affects drainage and substrate stability; and aspect determines sun exposure, which can impact basking behavior and habitat selection (Owen 1989). By integrating both climatic and topographic variables, we aimed to capture the full range of abiotic factors shaping the king cobra's current and future habitat suitability.

Although land cover and vegetation types are known to influence snake distribution by providing shelter, influencing prey availability, and affecting human disturbance, we were unable to include these variables in the present model due to the lack of high‐resolution, spatially consistent land cover data compatible with the bioclimatic layers. This limitation is acknowledged and discussed further in the manuscript, and we recommend that future modeling efforts incorporate land cover and habitat structure data for improved prediction accuracy.

### 2.5 Species Distribution Modeling

Species distribution modeling (SDM) was conducted using the BIOMOD2 package in R, which integrates multiple algorithms for ensemble modeling (R Core Team 2021; Thuiller et al. 2009, 2016). We used 10 SDM algorithms, which were generalized additive models (GAMs), generalized linear models (GLMs), generalized boosted regression model (GBM), multiple adaptive regression splines (MARS), random forest (RF), surface range envelope (SRE), classification tree analysis (CTA), flexible discriminant analysis (FDA), artificial neural network (ANN), and maximum entropy (MAXENT). Based on model evaluation using true skill statistics (TSS), only RF, Maxent, and GLM, with mean TSS values exceeding 0.6, were retained for the final ensemble model due to their superior predictive performance (Allouche et al. 2006; Thuiller et al. 2016). These algorithms are well recognized for their ability to handle complex ecological relationships. The dataset was split into training (80%) and testing (20%) subsets, following standard SDM practices (Elith et al. 2006; Phillips et al. 2006). To enhance robustness, 10,000 pseudoabsence points were randomly generated in environmentally unsuitable areas (Barbet‐Massin et al. 2012). Model performance was assessed using the area under the curve (AUC) and TSS metrics, with TSS > 0.7 indicating high accuracy. An ensemble model was constructed using a weighted mean approach, prioritizing algorithms with high TSS scores (Marmion et al. 2009). To assess the land‐use composition of climatically suitable habitats for the king cobra, we overlaid the thresholded binary habitat suitability map—derived from ensemble species distribution models and thresholded using TSS—onto the Sentinel‐2 land‐use/land‐cover (LULC) dataset at 10‐m resolution (Karra et al. 2021). This was done by performing a spatial intersection, wherein each pixel identified as suitable habitat was matched with its corresponding land cover class from the Sentinel‐2 raster. We then calculated the proportion of suitable habitat area within each land cover category. This approach enabled us to quantify how climatically suitable areas are distributed across different land‐use types, such as forest, cropland, grassland, and built‐up areas.

### 2.6 Anthropogenic Mortality of King Cobra and Trends of Conflict

Out of a total of 553 recorded occurrences of king cobras, 94 cases involved individuals that were either injured or deceased. To analyze the spatial distribution of risk, we created a binary matrix where 0 represented instances where king cobras were not injured or deceased, and 1 represented cases where they were. This matrix served as the foundation for generating a risk heatmap. To visualize the spatial patterns of king cobra mortality and injury, we utilized the Inverse Distance Weighting (IDW) interpolation method in ArcGIS, following the approaches outlined in Baral et al. (2022) and Kunwar et al. (2023). IDW is a deterministic spatial interpolation technique that assigns greater weight to data points closer to the location being estimated. This method interpolates values by computing a moving average of nearby points, with weights inversely proportional to the distance between the sampled points and the interpolated location, as described by Shepard (1968).

The IDW approach assumes that the influence of a given data point diminishes with increasing distance, making it particularly suitable for visualizing localized patterns of risk. The resulting heatmap highlights spatial variability in king cobra mortality and injury risk across the study area, providing insights into potential hotspots and areas requiring focused conservation efforts.

## 3 Results

### 3.1 Contribution of Variables and Model Performance

**FIGURE 2:**
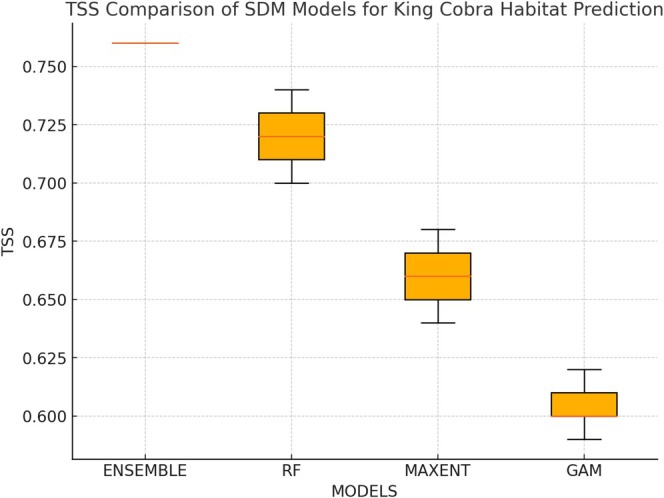
Boxplot representing the accuracy of the models used in BIOMOD2. The ensemble method yielded more accurate predictions than did the single‐algorithm models: random forest (RF), maximum entropy (MAXENT), and the random generalized additive model (GAM). The upper box limit, midline, and lower box limit represent the lower quartile (Q1), the median, and the upper quartile (Q3), respectively. The whiskers represent the extension of the box to the minimum and maximum values that fall within 1.5 times the interquartile range, and any values outside this range are outliers, which are represented by red dots. TSS, true skill statistics.

Among the 10 SDM algorithms explored, GAM, Maxent, and RF demonstrated the highest predictive performance, each achieving a mean TSS > 0.60 and AUC > 0.80, indicating robust model accuracy (Figure 2). The ensemble model, constructed from these three top‐performing algorithms, achieved the best overall performance with a TSS value of 0.76, highlighting its reliability for habitat suitability predictions (Figure 2).

**FIGURE 3:**
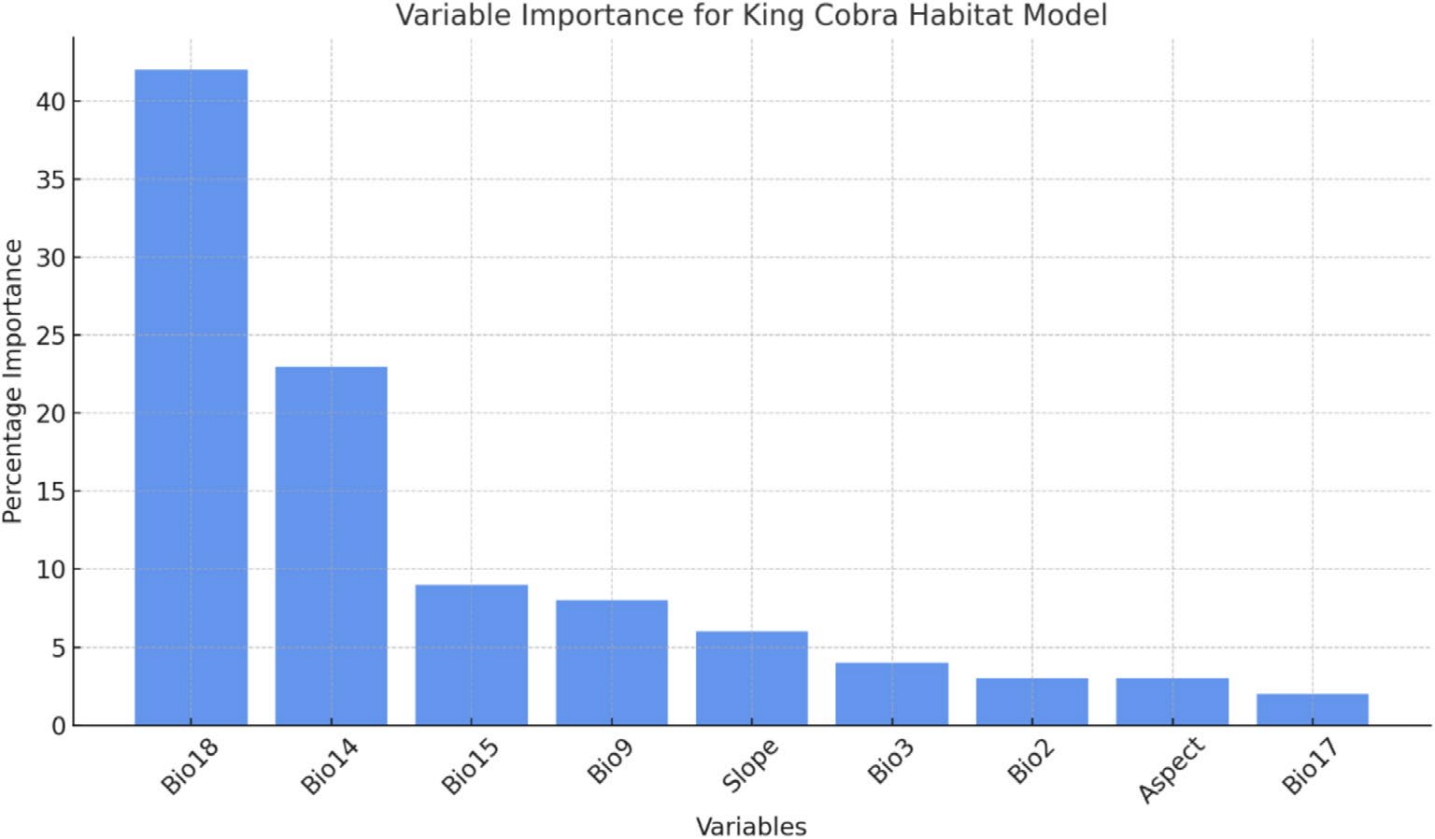
Importance of variables for generating the ensemble model for king cobra habitat.

**FIGURE 4:**
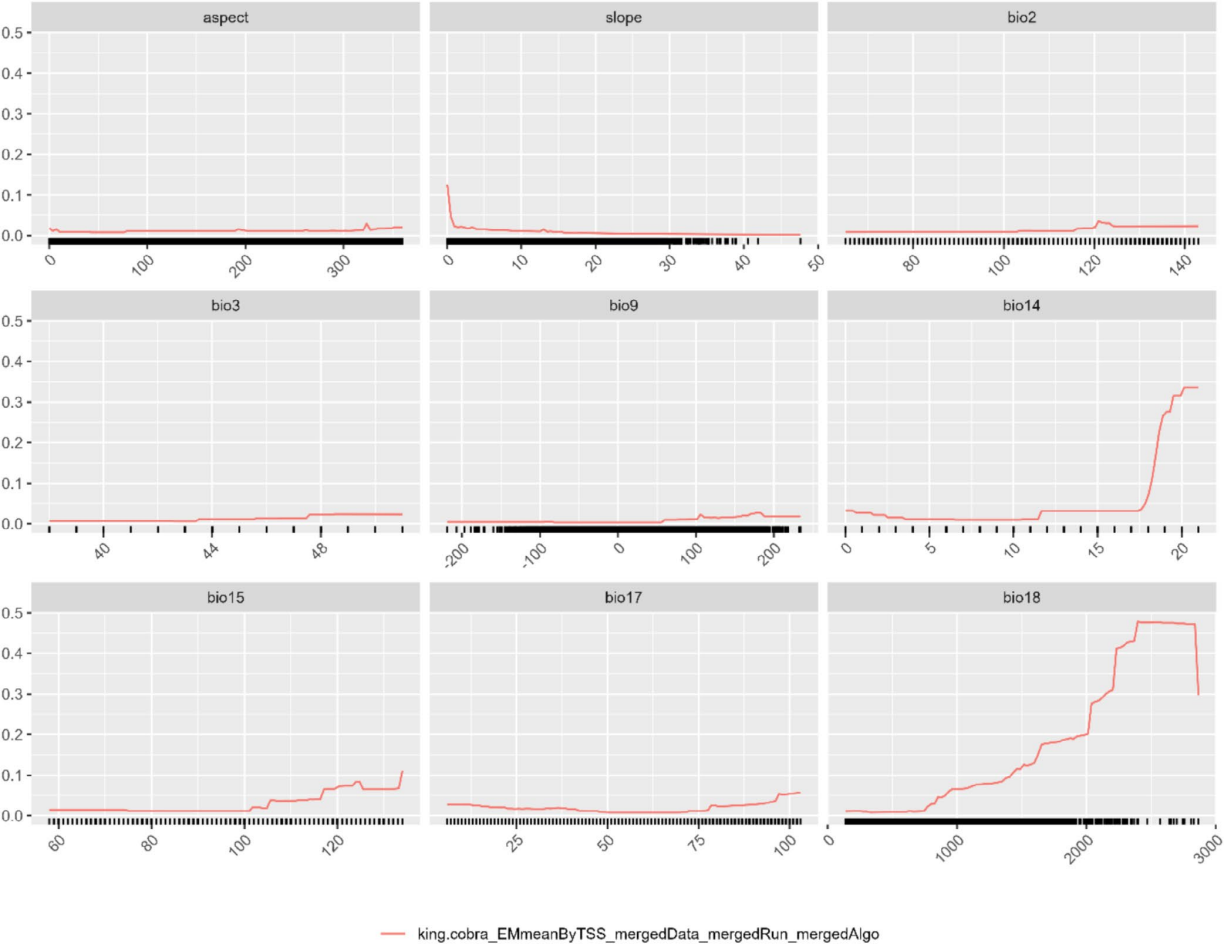
Response curves of king cobra (red line) to different bioclimatic variables viz. aspect, slope, bio2, bio3, bio9, bio14, bio15, bio17, and bio19, respectively, from top left to bottom right, respectively.

Among the entire variable, eight variables employed in the SDM (Figure 3), bio 18 (precipitation of warmest quarter) emerged as the paramount determinant (with the highest percentage contribution) for the ensemble model at 42%; followed by bio 14 (precipitation of driest month) at 23% for king cobra (Figure 4).

### 3.2 Current Distribution and Suitable Habitat Availability of the King Cobra in Nepal

Our models for the king cobra predicted that currently 23,702.62 km^2^ (16% of the total area of Nepal) is climatically suitable habitat (Table 2). Among the physiographic regions, the Siwalik constituted the largest suitable habitat (42%), followed by the Middle Mountains (37.96%). Bagmati province encompassed the largest area (30%) of the total suitable habitat, followed by Gandaki (25.04%) (Table 3). Central Nepal harbored the largest habitat patches compared with western and eastern Nepal, which had very fragmented patches of suitable habitat.

**TABLE 2:** Current and projected future suitable habitats for the king cobra in Nepal under climate change scenarios.

Species	Year	Area	% out of total area of Nepal	Habitat (within PA)	% Habitat within PA	Loss %
King cobra	Present	23,702.62	16%	3888.33	45%	
	2050	18,748.10	13%	2557.67	30%	21%
	2070	16,989.69	12%	2196.42	25%	9%

**TABLE 3:** Projected suitable habitat area (in km^2^) for the king cobra within each province of Nepal under the present and future climate scenarios (2050 and 2070) based on the SSP2‐4.5 scenario.

Provinces	Present	2050	2070
Koshi	3850.16	7121.02	6582.41
Madhesh	3685.21	2092.55	876.51
Bagmati	7311.19	5177.10	4234.96
Gandaki	5935.10	3515.16	4513.42
Lumbini	2690.38	767.15	612.15
Karnali	148.20	61.18	96.14
Sudurpaschim	82.39	13.94	74.10
Total	23,702.62	18,748.10	16,989.69

*Note:* The table shows expected spatial shifts in habitat suitability across provinces, with a general declining trend over time.

Of the total suitable habitat for king cobras, only 11.06% (2622.12 km^2^) is within the protected area (PA) system, with the largest portions in Chitwan National Park (32.14%), Annapurna Conservation Area (22.17%), and Parsa National Park (19.19%) (Table 4). Within the PA system, 3688.66 km^2^ of habitat is utilized, comprising 2622.12 km^2^ in core areas and 1066.20 km^2^ in buffer zones. Chitwan National Park and its buffer zone account for the highest coverage, followed by Annapurna Conservation Area and Parsa National Park (Table 4).

**TABLE 4:** Current and projected suitable habitats (in km^2^) for the king cobra within core and buffer zones of Nepal's protected areas under present, 2050, and 2070 climate scenarios (SSP2‐4.5).

Protected area		Present		2050		2070	
		Core area	Buffer zone	Core area	Buffer zone	Core area	Buffer zone
Conservation area (CA)							
1	Annapurna CA	581.44	—	605.75	—	621.37	—
2	Kangchenjunga CA	0.53	—	3.10		3.15	
3	Manaslu CA	13.83	—	2.60		2.07	
4	Gaurishankar CA	285.01	—	665.88		665.92	
5	Api Nampa CA	3.82	—				
6	Blackbuck CA	0.09		0.09		0.09	
National parks (NP)							
7	SheyPhoksundo NP	—	—				
8	Langtang NP	69.78	276.11	83.58	258.60	38.73	96.42
9	Sagarmatha NP	—	3.89	1.00	14.78	3.06	18.72
10	Shivapuri NP	90.55	—	87.58		67.28	
11	Chitwan NP	842.84	434.14	186.37	52.40	208.80	58.22
12	Makalu Barun NP	12.60	49.13	47.33	184.13	61.61	232.03
13	Rara NP						
14	Khaptad NP						
15	Banke NP	5.65	13.20	4.20	10.29	4.68	13.86
16	Bardia NP	153.43	18.76	25.59	1.86	32.56	2.57
17	Parsa NP	503.35	232.85	10.14	36.14	10.44	31.72
18	Suklaphanta NP	2.12	0.92		0.78	1.54	0.78
Hunting reserve (HR)							
19	Dhorpatan HR						
Wildlife reserves (WR)							
20	Koshi Tappu WR	57.08	37.19	142.39	133.11	7.72	13.07
	Total	2622.12	1066.20	1865.59	692.08	1729.03	467.40
	Grand total	3688.33		2557.67		2196.42	

*Note:* The table distinguishes habitat availability across conservation areas (CA), national parks (NP), and other protected zones, highlighting potential future shifts and the conservation importance of individual sites.

**FIGURE 5:**
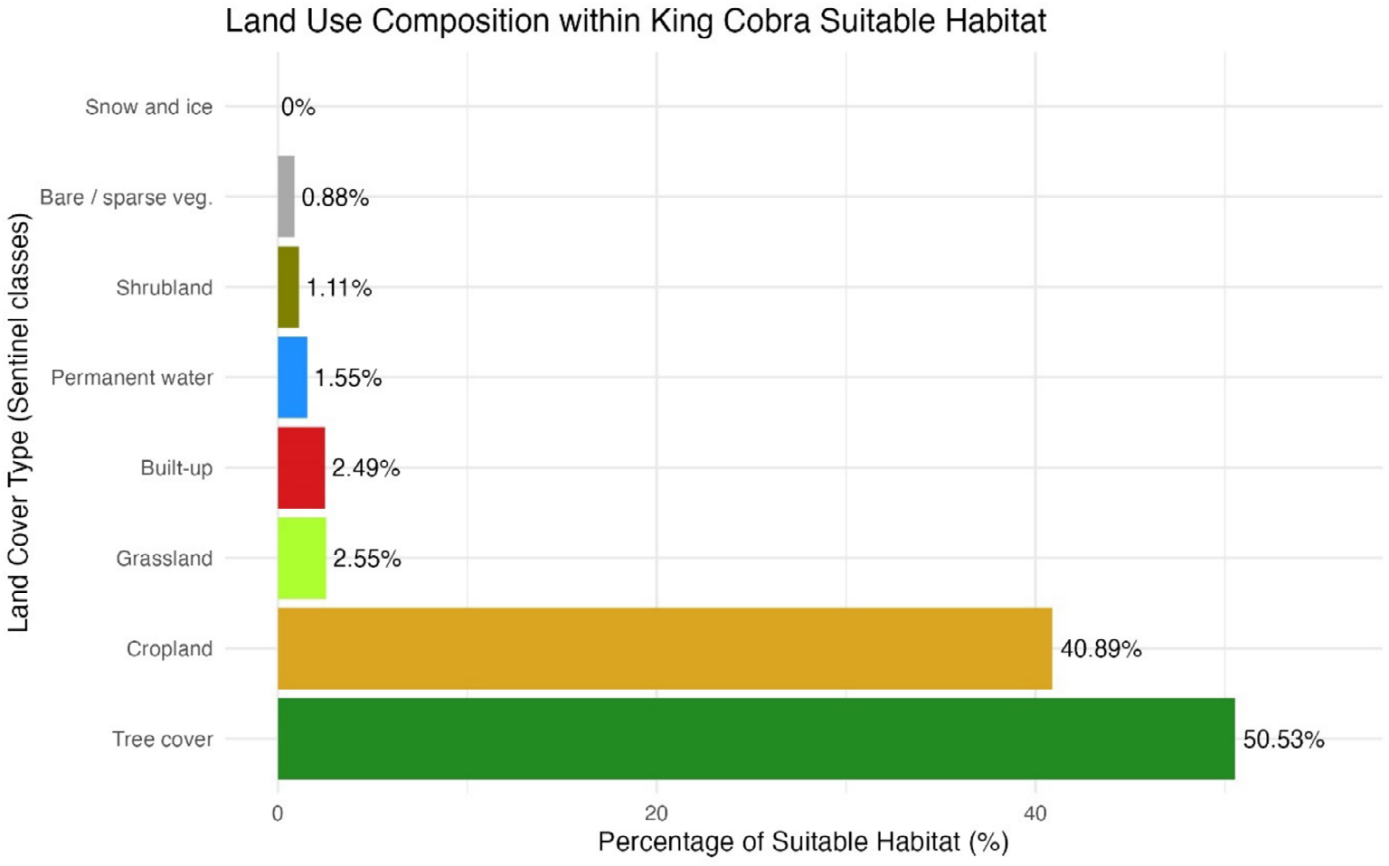
Proportion of land cover types across the current climatically suitable range of king cobra.

Across the current climatically suitable range, tree‐covered pixels dominate, accounting for 50.53% of all suitable habitat, followed by cropland at 40.89%. Open habitats make up only small fractions: grassland 2.55%, built‐up areas 2.49%, permanent water bodies 1.55%, shrubland 1.11%, and bare/sparse vegetation 0.88%; no suitable pixels coincided with snow‐and‐ice classes (Figure 5).

### 3.3 Future Distribution and Range Change

The suitable habitat for king cobra in Nepal is projected to experience substantial reductions and shifts under future climate scenarios. Modeling under the SSP2‐4.5 scenario predicts that by 2050 and 2070, 13% (18,748.10 km^2^) and 12% (16,989.69 km^2^) of Nepal's total area, respectively, will remain suitable for the species. This represents a 13% habitat loss by 2050 and a 12% loss by 2070 relative to the current distribution. Within protected areas (PAs), the suitable habitat is expected to decrease significantly, from 30% of the current PA coverage to 21% by 2050 and further to 9% by 2070 (Table 2).

**FIGURE 6:**
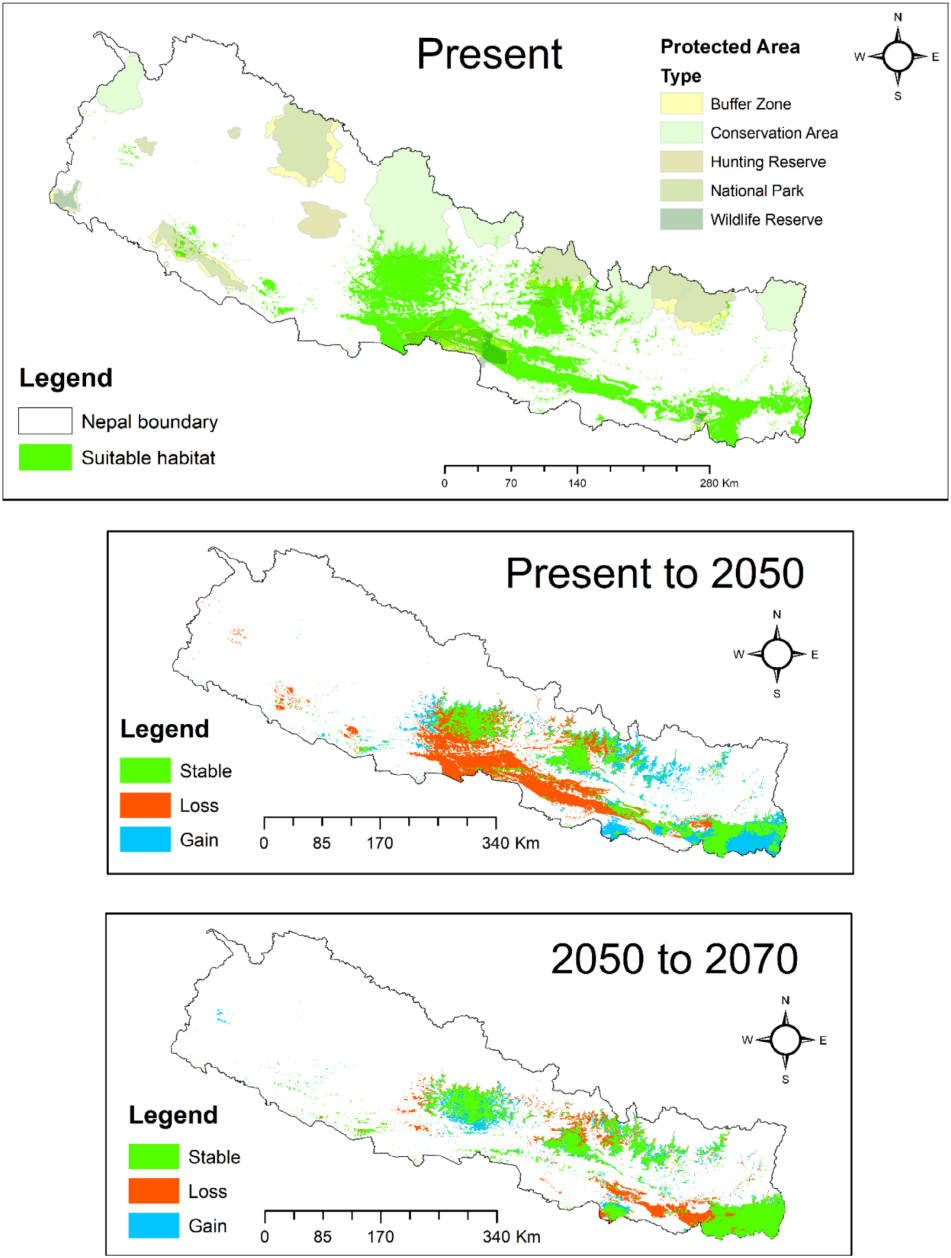
Projected spatial changes in suitable habitat for the king cobra in Nepal from the present to 2070 under the SSP2‐4.5 climate scenario. Green areas represent stable habitat (suitable in both time periods), blue indicates habitat gain (newly suitable areas), and orange shows habitat loss (currently suitable areas that become unsuitable in the future). SSP refers to Shared Socioeconomic Pathway.

Future projections indicate that habitat stability, loss, and gain will vary regionally. The model predicts potential habitat gains primarily in the eastern parts of Nepal, with some of these areas located outside the current PA boundaries. In contrast, significant habitat loss is anticipated in central Nepal, particularly within Bagmati and Gandaki Provinces, including areas around Chitwan National Park, Parsa National Park, and districts such as Kathmandu and Kaski (Figure 6). These findings underscore the urgent need for adaptive conservation strategies to mitigate habitat loss and ensure the persistence of king cobra populations in Nepal.

### 3.4 Anthropogenic Mortality of King Cobra and Trends of Conflict

**FIGURE 7:**
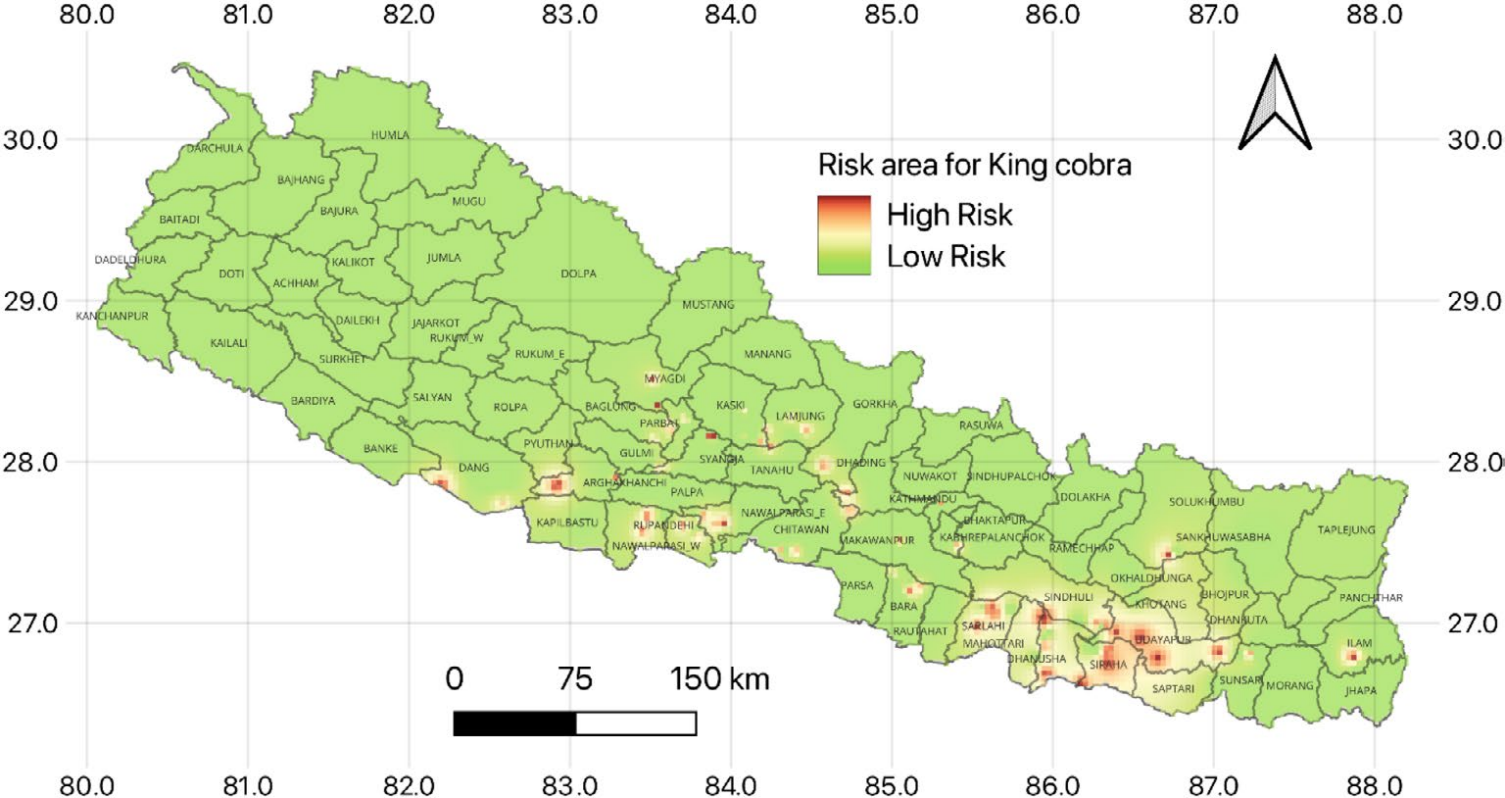
Inverse distance weighted (IDW) heatmap illustrating spatial variation in risk levels for king cobras based on recorded injury and mortality data. Green areas indicate low‐risk zones, yellow represents medium risk, and red signifies high‐risk zones, highlighting potential conflict hotspots and conservation priority areas.

Between 2000 and 2024, a total of 94 king cobra deaths were recorded across 29 districts in Nepal. Lowland districts, such as Dang, Arghakhanchi, Rupandehi, Nawalparasi (East and West), Chitwan, and eastern districts like Sarlahi, Mahottari, Dhanusha, Sindhuli, Siraha, Udayapur, Saptari, and Makwanpur, exhibited a higher likelihood of mortality. In the mid‐hill regions, districts including Myagdi, Kaski, Lamjung, Dhading, Parbat, and Sankhuwasabha were identified as high‐risk areas (Figure 7). Most mid‐hill districts were categorized as medium‐risk zones, while the high mountain and Himalayan regions exhibited a much lower probability of king cobra mortality. The heatmap analysis indicated eastern Nepal as the primary hotspot for king cobra mortality, underscoring the importance of targeted conservation measures in these areas (Figure 7).

## 4 Discussion

Our research employed ecological niche modeling (ENM) to evaluate the distribution of king cobras in Nepal under both current and future climate conditions. ENM is widely regarded as a reliable approach for forecasting species distributions, especially in relation to climate change and habitat suitability (Ashrafzadeh et al. 2022; Baral et al. 2024; Rezaei et al. 2022). The ensemble model used in our study achieved a high true skill statistic (TSS) value of 0.76, demonstrating its effectiveness in accurately identifying areas with potential habitat suitability for this species.

### 4.1 Important Variables

This study revealed that among the variables considered, eight were employed in the species distribution modeling (SDM). Of these, bio 18 (precipitation of the warmest quarter) emerged as the most significant determinant, contributing 42% to the ensemble model, followed by bio 14 (precipitation of the driest month) with a 23% contribution (Figure 3). These two precipitation‐related variables exhibited the highest positive association with the potential distribution of *Ophiophagus hannah* .

This strong association may be explained by the impact of precipitation and temperature changes on food availability during dry seasons (Dhakal et al. 2014). Altered precipitation patterns indirectly affect reptiles by influencing their habitats and food resources (Araújo et al. 2006). Such changes can lead to reduced body size, slower growth rates, and diminished lifetime fitness (Brown and Shine 2007; Loehr et al. 2007). Additionally, increased temperatures and lower humidity may promote color polymorphism (Harkey and Semlitsch 1988), potentially impacting their ability to evade predators or catch prey. Environmental changes could also impair visual sensitivity (Aho et al. 1988), reducing king cobras' capacity to detect predators, locate food, and find mates. As ectothermic organisms, reptiles like the king cobra depend on external heat sources to regulate their body temperature. Consequently, climate plays a critical role in shaping their distribution and abundance (Zug 1993). This dependency was evident in our results, where bioclimatic variables were key predictors of the king cobra's distribution in Nepal. Under future climate scenarios (SSP2‐4.5), our projections for 2050 and 2070 indicate an initial decline in highly suitable habitats for *O. hannah* , followed by an expansion by 2070. This finding aligns with observations by Mukherjee et al. (2024), who reported similar patterns across Asia for the periods 2041–2060 and 2081–2100 under SSP5‐8.5. For ectothermic species, habitat expansion under rising temperatures may result from improved thermal conditions that enhance habitat suitability (Precht et al. 1973).

However, global warming poses a severe threat to biodiversity, endangering many plant and animal species (Sapkota et al. 2021). Furthermore, Araújo and Pearson (2005) emphasized that reptiles often exhibit limited capacity to rapidly adapt their distributions to changing climatic conditions. Consequently, rising average temperatures may profoundly impact the spatial distribution, physiological processes, reproductive biology, and behavior of king cobras (Dunham 1993; Grant and Porter 1992).

### 4.2 Current Distribution, Suitable Habitat Availability, and Protected Area Suitability for King Cobras in Nepal

Our study highlights the restricted habitat availability for king cobras in Nepal, with only 16% of the total land area identified as climatically suitable. These habitats are primarily concentrated in the central and eastern Siwalik and Terai regions, extending into the Middle Mountains. The Siwalik region accounts for the largest proportion of suitable habitat (42%), followed by the Middle Mountains (37.96%). Among provinces, Bagmati encompasses the largest share of suitable habitat (30%), followed by Gandaki (25.04%). Central Nepal features the most extensive contiguous patches of habitat, while the western and eastern regions exhibit more fragmented distributions. Remarkably, our study documented the presence of king cobras in 13 new districts, significantly expanding their known range to 56 districts across Nepal. This includes Banke in the Siwalik region, 11 districts in the mid‐hills (Baitadi, Pyuthan, Bhaktapur, Bhojpur, Dhading, East Rukum, Khotang Nuwakot, Ramechhap, Sindhupalchok, Tanahu), and 2 in the high mountains (Bajhang and Jajarkot). These findings enhance the understanding of king cobra distribution and underscore the species' adaptability to diverse ecological zones.

Using an ensemble modeling approach with 553 occurrence points, our study provided a refined estimate of climatically suitable habitat, compared to previous studies. For instance, Sapkota et al. (2021), using only 131 occurrence points, estimated 27.93% of suitable habitat. Our larger dataset allowed for more precise and realistic predictions, reducing overestimations. Additionally, our findings align with Baral, Yadav, et al. (2019) and Baral, Subedi, and Yadav (2019), who reported king cobra sightings, live nests, and mating records, particularly in the southern Annapurna Conservation Area (ACA). Within Nepal's protected area (PA) network, Chitwan National Park (CNP), Annapurna Conservation Area (ACA), and Parsa National Park (PNP) host the largest proportions of suitable habitat, accounting for 32.14%, 22.17%, and 19.19%, respectively. These PAs are well‐known biodiversity hotspots, offering critical habitats for king cobras. Their proximity to rivers and tributaries enhances food availability, particularly for snake‐dependent predators like the king cobra. The southern parts of Gandaki and Bagmati provinces, characterized by numerous water sources and a diverse prey base, were identified as highly suitable areas for the species. However, only 11.06% of the total suitable habitat lies within the PA system. This highlights the need for conservation efforts to extend beyond PAs. Our findings emphasize the critical role of habitat corridors, buffer zones, and nonprotected landscapes in maintaining ecological connectivity and ensuring the survival of species like the king cobra.

However, only 11.06% of the total suitable habitat lies within the PA system, highlighting the limitation of relying solely on PAs for conservation of wide‐ranging species like King Cobra. PAs are important for biodiversity conservation, but their small size and isolation make them insufficient to uphold viable populations of various wildlife species (e.g., King Cobra) (Naughton‐Treves et al. 2005; Shrestha et al. 2010). This highlights the need for conservation efforts to extend beyond PAs. So, the forested areas beyond PAs are crucial for King Cobra's conservation, linking up fragmented habitats. For mammals, the establishment of Community‐Based Forest Management in ecological bottlenecks and critical corridors, as suggested by Karanth et al. (2010), can greatly improve habitat connectivity if replicated for King Cobras as well. These corridors serve as refuges for biodiversity, offering alternative habitats and ecological connectivity that are vital for vulnerable species like King Cobra while mitigating climate change impacts (WWF 2013). Our findings emphasize the critical role of habitat corridors, buffer zones, and nonprotected landscapes in maintaining ecological connectivity and ensuring the survival of species like the King Cobra.

In conclusion, the king cobra's distribution and habitat suitability in Nepal demonstrate the interplay between natural ecosystems and human landscapes. Conservation strategies should focus on protecting habitats within PAs and addressing habitat fragmentation outside these areas. Efforts to conserve water resources, enhance habitat corridors, and mitigate climate change impacts will be essential to secure the future of this apex predator.

### 4.3 Future Distribution and Range Change

The king cobra, as a habitat generalist, exhibits remarkable ecological flexibility, enabling it to adapt to a variety of habitats ranging from pristine natural areas to human‐dominated landscapes, including agricultural fields (Marshall et al. 2019, 2020; Rao et al. 2013; Stuart et al. 2012a). This adaptability suggests that the species may be capable of adjusting to changing environmental conditions, potentially expanding its range. Our model results support this notion, revealing a projected decline in suitable habitat by 2050; followed by a potential expansion by 2070 under the SSP2‐4.5 climate scenario.

The ensemble modeling results predict a 13% reduction in suitable habitat for the king cobra by 2050 and a 12% reduction by 2070 compared with the current distribution, which encompasses 16% of Nepal's total area. Within protected areas (PAs), the loss is more pronounced, with suitable habitat declining from 30% of the current PA coverage to 21% by 2050 and further to 9% by 2070. These findings align with other studies of species distribution under climate change scenarios, such as those by Mukherjee et al. (2024) on king cobra in Asia, as well as Baral et al. (2024) and Budhathoki et al. (2023) on bear species and Asian elephants, respectively, which show similar patterns of initial habitat loss followed by range expansion.

The significant loss of suitable habitat within PAs may be attributed to conservation priorities focusing on mega‐faunal species such as the Red Panda, Bears, Bengal Tiger, Asian Elephant, One‐horned Rhinoceros, and Wild Buffalo. These priorities often overshadow the needs of smaller species like the king cobra, potentially leading to declines in prey populations crucial for the species' survival. The model results support this, as new king cobra distribution records largely emerge from the mid‐hill region, an area not traditionally considered the species' most suitable habitat, which is generally found in the lowland regions of Nepal. This shift in distribution suggests an altitudinal movement uphill, likely driven by changes in habitat quality or prey availability in the lowlands. While the lowland regions of central Nepal still offer suitable habitat, the expansion of king cobra populations into mid‐hill districts, such as Baitadi, Pyuthan, and Bhojpur, indicates an upward range shift. This trend is consistent with studies in other species, including the findings of Mi et al. (2024), which highlighted how climate factors like temperature and precipitation can limit the distribution of reptile species, influencing their spatial range.

King cobras are opportunistic in utilizing habitats based on resource availability, with prey populations likely being a critical determinant of their distribution (Marshall et al. 2020). Our findings corroborate this behavior, as evidenced by the 11 new district records from the mid‐hills. These records suggest that king cobras are increasingly occupying areas outside their traditional lowland habitats in response to shifting resource dynamics. Notably, while the species is expanding its range across all districts in the Terai region, the Kapilbastu district remains an exception, possibly due to the ongoing uphill movement from the Terai to the Siwalik and mid‐hills.

The model further suggests that habitat stability, loss, and gain will vary regionally. Significant habitat gains are predicted in the eastern parts of Nepal, particularly outside the current PA boundaries. Conversely, habitat loss is expected to be most pronounced in central Nepal, specifically in the Bagmati and Gandaki Provinces, as well as in areas surrounding Chitwan National Park, Parsa National Park, and districts like Kathmandu and Kaski. These findings emphasize the need for targeted conservation strategies that address the habitat requirements of king cobras both inside and outside protected areas. As climate change continues to reshape suitable habitats, the protection of these corridors, along with the preservation of prey species, will be crucial to ensuring the long‐term survival of king cobra populations in Nepal.

### 4.4 Anthropogenic Mortality of King Cobra and Trends of Conflict

King cobras face significant extinction risks due to several life‐history traits, including their specialized diet (Jones et al. 2020), large body size (Marshall et al. 2018), and slow rate of maturity (Böhm et al. 2016; Todd et al. 2017). Additionally, their reliance on large home ranges, often within agricultural landscapes, makes them particularly vulnerable to human–wildlife conflict and human‐mediated threats, such as habitat encroachment and direct persecution (Marshall et al. 2018, 2019, 2020). These ecological and human behavioral factors make conservation efforts for king cobras particularly challenging in areas with high human activity, such as Nepal and other parts of their range. Our study confirms that these factors contribute to an increasing trend in king cobra mortality over the past 25 years, highlighting the growing conflict between human activities and the survival of the species.

In a prior study, Devkota et al. (2021) documented 50 king cobra mortality cases across 20 districts in Nepal. In comparison, our study recorded 44 additional mortality cases from 29 districts, significantly broadening the geographic scope of reported deaths. Notably, 11 of these additional cases were reported from 9 new districts—Bhojpur, Dang, Ilam, Lalitpur, Nawalparasi, Palpa, Pyuthan, Solukhumbu, and Sunsari—while the remaining cases occurred in districts previously documented. These findings highlight the escalating human–wildlife conflict and increasing anthropogenic pressures on king cobra populations in Nepal.

The study found that lowland districts and eastern regions, such as Madhesh and Lumbini provinces, exhibited higher mortality rates, likely due to lower literacy rates and high levels of human activity. In these regions, literacy rates are 63.5% in Madhesh and 78.1% in Lumbini (National Population and Housing Census 2021), which could contribute to a lack of awareness about the species, increasing the risk of conflict. Conversely, the mid‐hill regions, such as Gandaki and Bagmati provinces, had medium‐risk zones, with high literacy rates (81.7% and 82.1%, respectively) but also elevated human activity. The high mountain and Himalayan regions were identified as lower‐risk zones, likely due to less human activity and lower population density compared to other regions of Nepal. These findings suggest that king cobra mortality is not only influenced by human activities but also by the degree of local awareness and education. The spatial expansion of mortality risk zones, particularly in the lowlands and mid‐hills, could increase further due to the ongoing altitudinal shift in king cobra distribution (Mukherjee et al. 2024). As king cobras expand their range, new regions may experience heightened mortality risk, necessitating targeted conservation efforts in both newly identified hotspots and areas where mortality has not yet been reported but is expected.

Despite being a secretive and elusive species, the king cobra is often perceived as a threat due to its size and potential danger; even though human‐bite incidents are rare in Nepal. This perception is rooted in a lack of knowledge about the species and its conservation status, highlighting the need for increased public awareness and education. The increasing trend of king cobra mortality may be linked to a lack of information and understanding, emphasizing the importance of conservation campaigns and outreach efforts. Encouragingly, recent mortality trends indicate that conservation activities and media outreach by governmental agencies and NGOs, such as the Snake Conservation Society Nepal, have helped mitigate mortality. Higher literacy rates and increased public awareness through conservation messages have contributed to a decrease in mortality rates over time, suggesting that local communities are gradually adopting a more coexistence‐based approach. In conclusion, while king cobra mortality remains a concern, our findings suggest that targeted conservation efforts, including public education and awareness campaigns, have had a positive impact on the species' conservation in Nepal. However, continued efforts are required, particularly in regions with low literacy rates and high human activity, to reduce mortality and foster coexistence. The results from our study emphasize the need for adaptive conservation strategies that address both the ecological needs of king cobras and the socioeconomic factors contributing to human–wildlife conflict.

## 5 Implications of the Findings for Prevention and Management

Despite being a secretive and elusive species, the king cobra is often perceived as a threat due to its size and potential danger, even though human‐bite incidents are rare in Nepal. This perception is rooted in a lack of knowledge about the species and its conservation status, highlighting the need for increased public awareness and education. The increasing trend of king cobra mortality may be linked to a lack of information and understanding, emphasizing the importance of conservation campaigns and outreach efforts. Encouragingly, recent mortality trends indicate that conservation activities and media outreach by governmental agencies and NGOs, such as the Snake Conservation Society Nepal, have helped mitigate mortality. Higher literacy rates and increased public awareness through conservation messages have contributed to a decrease in mortality rates over time, suggesting that local communities are gradually adopting a more coexistence‐based approach.

In light of these findings, our study underscores the importance of conservation activities that include public outreach and education, especially in regions where awareness is currently lacking. The heatmap generated by our study provides a valuable tool for identifying and monitoring conflict zones. Targeted conservation efforts, such as community education about King Cobra ecology and behavior, could reduce fear and promote coexistence. This proactive approach could ultimately help reduce King Cobra mortality and foster a more sustainable coexistence between humans and wildlife in Nepal.

## 6 Limitations of the Study

Although our 1‐km ensemble models and nationwide mortality heatmap provide the most detailed picture yet of king cobra conservation needs in Nepal, several caveats remain: (i) the 553 occurrence records, while unprecedented, still under‐represent remote areas where this cryptic snake is seldom detected, so some sampling bias is inevitable; (ii) future projections incorporate only climate change because comparable, downscaled land‐use/land‐cover layers for 2050–2070 are not yet available, meaning habitat shifts driven by deforestation or urban expansion are unmodelled; (iii) we relied on a single, policy‐relevant emissions pathway (SSP2‐4.5), so follow‐up work should bracket best‐ and worst‐case scenarios (SSP1‐2.6 and SSP5‐8.5) to test corridor robustness; (iv) broad BIOCLIM averages cannot capture fine‐scale features such as canopy shade, leaf‐litter moisture, or monsoon timing that strongly influence snake activity; and (v) the IDW conflict surface is restricted to observed mortalities—forward‐looking risk maps will require scenario‐based models linking projected roads, population growth, and land‐use change to snake behavior. These limitations do not diminish the current findings but highlight priority directions for refining future king cobra conservation assessments.

## 7 Conclusion

This study provides critical insights into the current distribution, suitable habitat, and anthropogenic mortality trends of the king cobra in Nepal. The habitat suitability models identified 16% of Nepal's total area as climatically suitable for the species, with significant regional variability. However, future projections under climate change scenarios indicate substantial habitat loss, particularly in central Nepal, while some areas in the east may experience habitat gains. Anthropogenic mortality has increased over the past 25 years, with a clear correlation between king cobra deaths and human activity, particularly in lowland and mid‐hill regions. The decreasing suitable habitat in recent years and mortality cases in newly known distribution areas suggest that the king cobra requires a specific conservation action plan in Nepal. This plan could help address the challenges posed by habitat loss and human–wildlife conflict, ensuring the conservation of this iconic and globally threatened species.

## Author Contributions

**Rishi Baral:** conceptualization (lead), data curation (lead), formal analysis (equal), investigation (lead), methodology (lead), project administration (lead), resources (lead), software (equal), writing – original draft (lead), writing – review and editing (lead). **Binaya Adhikari:** data curation (supporting), formal analysis (lead), methodology (supporting), software (lead), writing – original draft (supporting), writing – review and editing (supporting). **Abhisek Sapkota:** data curation (supporting), methodology (supporting), resources (supporting), writing – original draft (supporting), writing – review and editing (supporting). **Saroj Panthi:** data curation (supporting), formal analysis (supporting), methodology (supporting), writing – review and editing (supporting). **Chiranjeevi Khanal:** data curation (supporting), writing – review and editing (supporting). **Sandesh Gurung:** data curation (supporting), methodology (supporting). **Keshab Raj Sapkota:** data curation (supporting). **Naresh Subedi:** supervision (supporting), validation (supporting), visualization (supporting). **Bishwanath Rijal:** data curation (supporting), resources (supporting). **Raju Acharya:** data curation (supporting), supervision (supporting). **Michito Shimozuru:** conceptualization (supporting), supervision (equal), writing – review and editing (supporting). **Toshio Tsubota:** conceptualization (supporting), supervision (lead), validation (lead), writing – review and editing (supporting).

## Disclosure

I confirm that this manuscript contains entirely original work and has not been published elsewhere, either in whole or in part, in any format. The corresponding author has secured consent from all contributing authors for the submission of this manuscript. Regarding animal ethics, this declaration is not applicable. Additionally, all required permissions have been obtained from the relevant authorities where applicable.

## Conflicts of Interest

The authors declare no conflicts of interest.

## Data Availability

Supporting Information can be accessed via Dryad at the following link: https://doi.org/10.5061/dryad.ht76hdrsm.
